# Interpretable Deep Learning System for Identifying Critical Patients Through the Prediction of Triage Level, Hospitalization, and Length of Stay: Prospective Study

**DOI:** 10.2196/48862

**Published:** 2024-04-01

**Authors:** Yu-Ting Lin, Yuan-Xiang Deng, Chu-Lin Tsai, Chien-Hua Huang, Li-Chen Fu

**Affiliations:** 1 Department of Computer Science and Information Engineering National Taiwan University Taipei Taiwan; 2 Department of Emergency Medicine National Taiwan University Hospital and National Taiwan University College of Medicine Taipei Taiwan

**Keywords:** emergency department, triage system, hospital admission, length of stay, multimodal integration

## Abstract

**Background:**

Triage is the process of accurately assessing patients’ symptoms and providing them with proper clinical treatment in the emergency department (ED). While many countries have developed their triage process to stratify patients’ clinical severity and thus distribute medical resources, there are still some limitations of the current triage process. Since the triage level is mainly identified by experienced nurses based on a mix of subjective and objective criteria, mis-triage often occurs in the ED. It can not only cause adverse effects on patients, but also impose an undue burden on the health care delivery system.

**Objective:**

Our study aimed to design a prediction system based on triage information, including demographics, vital signs, and chief complaints. The proposed system can not only handle heterogeneous data, including tabular data and free-text data, but also provide interpretability for better acceptance by the ED staff in the hospital.

**Methods:**

In this study, we proposed a system comprising 3 subsystems, with each of them handling a single task, including triage level prediction, hospitalization prediction, and length of stay prediction. We used a large amount of retrospective data to pretrain the model, and then, we fine-tuned the model on a prospective data set with a golden label. The proposed deep learning framework was built with TabNet and MacBERT (Chinese version of bidirectional encoder representations from transformers [BERT]).

**Results:**

The performance of our proposed model was evaluated on data collected from the National Taiwan University Hospital (901 patients were included). The model achieved promising results on the collected data set, with accuracy values of 63%, 82%, and 71% for triage level prediction, hospitalization prediction, and length of stay prediction, respectively.

**Conclusions:**

Our system improved the prediction of 3 different medical outcomes when compared with other machine learning methods. With the pretrained vital sign encoder and repretrained mask language modeling MacBERT encoder, our multimodality model can provide a deeper insight into the characteristics of electronic health records. Additionally, by providing interpretability, we believe that the proposed system can assist nursing staff and physicians in taking appropriate medical decisions.

## Introduction

### Background

Emergency services are an essential aspect of the health care system in hospitals, and the demand for these services has increased exponentially in recent years. For instance, due to a rising number of elderly patients, a high volume of low-acuity patients waiting for the emergency department (ED), and limited access to medical resources in the community, it may take a long time for patients to receive medical treatment in the ED. Additionally, the situation has worsened with the shortage of experienced health care providers. In the ED, this can cause many severe clinical outcomes, such as delayed diagnosis, longer patient wait times, and increased mortality rates. Moreover, the patient and the standard health care operation procedure may be disturbed. Therefore, prioritizing ED visits and maintaining the regular operation of the health care system are essential.

Triage is the process of accurately assessing patients’ symptoms and providing them with proper clinical treatment in the ED. Patients are assigned different priorities depending on their vital signs and chief complaints, and the judgment description from the nursing staff [1]. Many countries have developed their triage process to stratify the clinical severity of patients and thus distribute medical resources. For instance, the US Emergency Severity Index (ESI), Canadian Triage and Acuity Scale (CTAS) [2], and Taiwan Triage Acuity Scale (TTAS) are designed to improve the triage prioritizing process [3-5]. In terms of personnel, hospitals employ dedicated nurses who have been certified by the authorities to undertake the triage process. It is also essential to maintain the quality of education, training, and evaluation of those professionals, which is more difficult nowadays with the increase in the complexity of emergency care and the increase in the number of patients visiting the ED nationwide [6]. Although many standardized scales have been adopted to improve the process, there are still some limitations of the current triage system [7-9]. Among these issues, the lack of capability to prioritize patients and assign patients to appropriate triage levels is the most serious problem. According to records collected in Taiwan from 2009 to 2015, 167,598 out of 268,716 (nearly 60%) visits in the ED were assigned to level 3 in the triage process. In addition, 5-level triage mainly relies on an experienced nurse’s diagnosis that is based on a mix of subjective and objective criteria. Any human judgement errors or even inaccurate measurements that occur during the triage assessment can severely affect the outcome.

### Related Work

#### Contextualized Word Embedding

A word vector is an attempt to mathematically capture the syntactic and semantic features of a word and represent its meaning simultaneously. Computers calculate how often words appear next to each other by going through a large corpus. For instance, with GloVe [10] or word2vector [11], the word can be projected into a high-dimensional vector for further tasks.

Although these traditional word embedding methods are easy to understand and simple to implement, some limitations still need to be addressed. For example, after applying word vectors, it would be tough to train systems equipped with the softmax function owing to a large number of categories. On the other hand, the GloVe word embedding involves a numeric representation of a word regardless of where the word occurs in the sentence and the different meanings the word may have. Hence, several language models have been proposed to address these limitations, including embeddings from language models (ELMo) [12], bidirectional encoder representations from transformers (BERT) [13], and generative pretrained transformer (GPT) [14]. These celebrated language models generate general contextualized sentence embeddings by using a large scale of unlabeled corpora.

Among these famous models, BERT is the most popular model commonly used in solving natural language processing (NLP) tasks. BERT is a language model trained bidirectionally, which means that as compared to single-direction language models, it can provide a more profound sense of language context and flow. Moreover, instead of predicting the next word in the sentence, BERT also uses a novel method called “mask language modeling” (MLM). This novel algorithm randomly masks the words and then predicts them. BERT relies on the transformer architecture; however, since BERT aims to generate a language representation model, it only uses the transformer encoder by stacking them up. Later, with the help of MLM and “next sentence prediction” (NSP), BERT can achieve significant performance on lots of NLP downstream tasks by further fine-tuning on specific domains.

#### Deep Learning for Tabular Data

In statistics, tabular data refer to data organized in a table. Within the table, the rows and columns represent observations and attributes for those observations, respectively. Although many domains like vision, NLP, and speech enjoy the benefit of deep learning models, tabular data using deep learning methods remain questionable. On the other hand, when it comes to handling tabular data, the traditional machine learning method dominates most of the benchmarks and is commonly used in competitions, such as Kaggle, around the world. The conventional machine learning methods include methods based on decision tree (DT) such as extreme gradient boosting (XGBoost) [15], category boosting (CatBoost) [16], and light gradient boosting machine (LightBGM) [17]. The strength of these DT-based methods is that their output is easy to understand and available to provide interpretability without requiring any statistical knowledge. However, there are still some limitations of DT-based methods. Among these limitations, the most serious is that DT-based methods do not allow efficient learning with image or text encoders. Hence, many experts turn to deep learning methods instead of DT-based methods. Deep learning models enable end-to-end learning for tabular data and have many benefits at the same time. First, they can achieve better performance in a bigger data set. Second, they can alleviate the need for feature engineering. Finally, they encode multiple data types efficiently, like images along with tabular data.

However, the shortcoming of most deep learning methods is that they cannot provide interpretability. Fortunately, researchers have been aware of the problem in recent years, and several deep learning models with interpretability have been proposed, such as TabNet [18], neural oblivious decision ensembles (NODE) [19], and TabTransformer [20].

#### Current Work in the Triage System

Although current triage systems, such as the ESI and TTAS, follow clear guidelines to assign patient acuity, it implicitly leaves room for clinician interpretation. Hence, the diagnosis still depends heavily on the judgment and experience of individual nursing staff. Several studies have shown that cognitive biases can influence clinical judgments [6]. In written case scenarios at multiple EDs, the average accuracies of nurses were 56.2%, 59.2%, and 59.6% in Taiwan, Brazil, and Switzerland, respectively [21]. In view of this, some studies [6,21,22] have turned to the use of artificial intelligence (AI) systems to assist with decision-making in triage. They also demonstrate the system’s effectiveness with higher accuracy from the assisted means.

Numerous studies have attempted to use traditional machine learning methods in their approaches. Choi et al [6] used 3 types of conventional machine learning methods, including logistic regression, random forest, and XGBoost, to predict the Korea Triage Acuity Scale (KTAS) level. They used patients’ chief complaints as categorical features, meaning that they assigned a key code to each symptom. Their best model using random forest achieved precision, recall, and area under the receiver operating characteristic curve values of 0.737, 0.730, and 0.917, respectively. Liu et al [22] used CatBoost as their model; however, the study focused on distinguishing the mis-triage of patients in levels 3 and 4 since they believed that the under-triage of critically ill patients could be life-threatening. Their model was able to reduce the life-threatening mis-triage rate from 1.2% to 0.9% prospectively. Ivanov et al [21] carried out a series of experiments to demonstrate the effectiveness of their novel idea “clinical natural language processing (C-NLP).” To cope with free-text data, C-NLP uses sentence tokenization, word tokenization, and part-of-speech tagging to extract the meaning behind free-text data. Their best model included C-NLP and XGBoost, and it was able to achieve an accuracy of 75.7%, which is 26.9% higher than the average nurse’s accuracy.

The previously mentioned studies [6,21,22] achieved great performance in dealing with triage-level problems; however, these methods still have some limitations. Our proposed model aims to address these limitations and alleviate them. Multimedia Appendix 1 presents comparisons between earlier work and our study in different aspects.

### Goal of This Study

Although the studies mentioned in the previous section successfully demonstrated that AI improved the triage system for predicting triage level, they unfortunately had some serious drawbacks. In this study, we attempted to overcome these drawbacks while developing an appropriate prediction system based on triage information, including demographics, vital signs, and chief complaints. We propose a system that can handle the collected heterogeneous data, including tabular data and free-text data. The proposed system is capable of providing precise suggestions for ED staff in hospitals, and it has interpretability for better acceptance by users. Moreover, it is applicable to real-world situations.

## Methods

### System Overview

**Figure 1:**
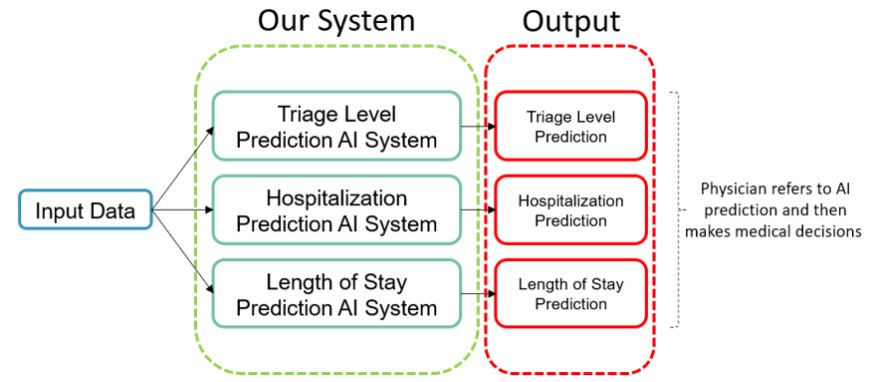
The proposed system comprising 3 subsystems that are responsible for different tasks. AI: artificial intelligence.

In this study, we have proposed a system comprising 3 subsystems, with each of them handling 1 task. As shown in Figure 1, these tasks include triage level prediction, hospitalization prediction, and length of stay prediction, which are important outcomes in the ED of a hospital. Since these subsystems are developed in a similar training process, we will first introduce the conceptual level of the typical training process of each model in each subsystem and then provide further information. Finally, we will show the detailed design of each model in each subsystem.

Our study focuses on establishing an effective and precise AI system to predict the criticality of patients waiting in the ED of hospitals. By leveraging a model trained on a data set where data labels include different scales, we look forward to developing a robust model that can provide more information to the physician and nursing staff. Moreover, to assist them in making precise medical decisions, our proposed system offers multiple prediction outcomes, including triage-level classification, hospitalization estimation, and length of stay.

**Figure 2:**
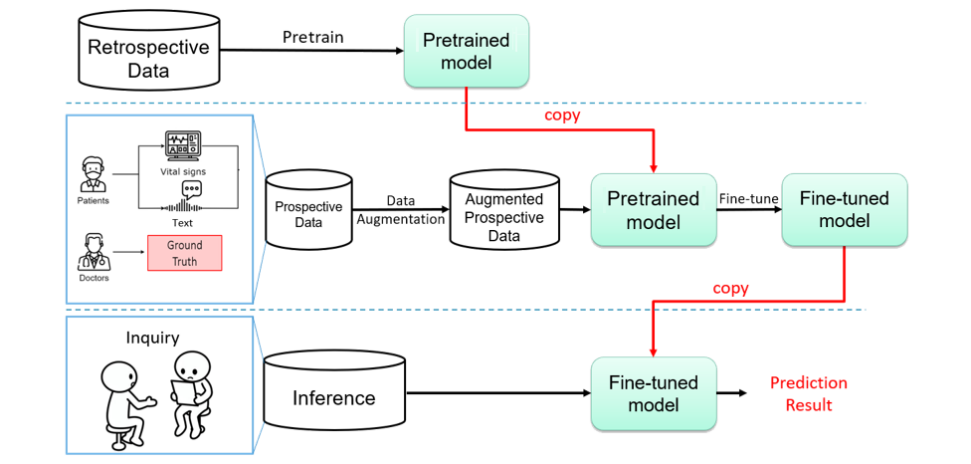
System flowchart.

The system flowchart is shown in Figure 2. The system can be divided into 3 stages: pretraining stage, fine-tuning stage, and testing stage. Additionally, 2 data sets were used in our study. One was the National Taiwan University Hospital (NTUH) retrospective data set, and the other was the NTUH prospective data set collected from May 26, 2020, till February 21, 2022. These 2 data sets will be elaborated in the following sections.

In the pretraining stage, a large amount of retrospective data were used to pretrain the encoders to learn the basic information of the medical data. In addition, the pretrained encoders were transferred to the second stage. In the fine-tuning stage, we used prospective data with golden labels to fine-tune the pretrained encoder. Therefore, when the diagnosis outcomes from the physician are treated as the ground truth label, the model is more applicable to real-world situations. Finally, in the testing stage, we implemented our system in the hospital and assessed the effectiveness of the system.

### Ethical Considerations

This study has been approved by the NTUH Institutional Review Board (201606072RINA, 201911054RINA, 202108090RINC).

### Data Preparation

#### NTUH Retrospective Data Set

The NTUH is a tertiary academic medical center that has almost 2400 beds and 100,000 emergency room visits per year. After receiving approval from the NTUH Institutional Review Board, we obtained the NTUH retrospective data set, which contained a total of 745,441 electronic health records (EHRs) of patients who visited the ED from the years 2009 to 2015. Since triage is the starting point of care for the ED, it is essential to ensure consistent and precise estimation of patients. The records were evaluated by dedicated personnel who were certified by the Taiwan Union of Nurses Association (TUNA), following a standard protocol.

**Figure 3:**
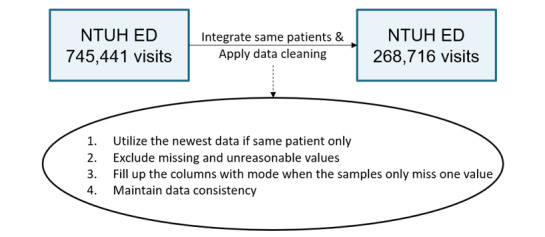
Preprocessing of the National Taiwan University Hospital (NTUH) retrospective data set. ED: emergency department.

As shown in Figure 2, in the first stage, we used the NTUH retrospective data set to pretrain our model. However, in the NTUH retrospective data set, we needed to unify the uncleaned data (Multimedia Appendix 2) initially as the members of the nursing staff have their own ways to record the estimation. We included all patients aged 20 years or older who attended the ED and excluded patients whose EHR data contained missing or unreasonable values. Unreasonable data had unreasonable values, which may have resulted from typing errors. For instance, the diastolic pressure and systolic pressure may be typed in reverse, or a nurse may accidentally omit a digit when entering values on the computer. In such a scenario, even though we may be able to infer the original intended values by examining individual data, we cannot consider this a correct sample for use. After data cleaning and merging, only 268,716 patients were enrolled in our program (Figure 3).

#### NTUH Prospective Data Set

Each patient who visits the ED will have a PDF document form generated (triage examination and evaluation record). These records are kept for the physician to make a diagnosis. The records comprise 2 types of information. The first is structural data, including patient demographics, triage information, and vital signs, and the second is textual data, including chief complaints, historical medical information, and drug allergy.

In general, it is impossible to directly use the aforementioned records to train the model, and thus, data preprocessing is needed to extract the data from the records. We used the PDFMiner library in Python code to extract the information from the document forms as “structural data” and applied a transformation function to generate “textual data.”

The information extracted from the forms and records can be divided into 2 groups: target prediction and patient feature. Detailed explanations of the patient features are provided in Table 1. On the other hand, the target ground truth contains 3 different tasks. The first task is triage level prediction, which is a 4-class classification problem, where the physician’s suggestion is considered (golden standard label that is obtained from the physician by observing the process of patient diagnosis) instead of the traditional triage level. A lower level indicates that the patient more urgently requires immediate attention. The second task is hospitalization prediction, which is a 2-class classification problem, where “0” represents that the patient needs to be discharged by the hospital and “1” represents that the patient needs to be admitted. The last task is length of stay, which is a 3-class classification problem, where “0” represents that the patient will stay in the ED for less than 6 hours, “1” represents that the patient will stay in the ED for 6 to 24 hours, and “2” represents that the patient will stay in the ED for more than 24 hours.

**Table 1:** Detailed explanation of structural variables.

Variable		Explanation
**Demographics**		
	Age	Patient age
	Sex	Patient gender
**Triage information**		
	Session	Patient arrival time
	Return in 24 hours	Number of times the patient revisited the ED^a^ in 24 hours
	Clinic visit mode	Patient arrival mode
	Work related	Whether the patient visited the ED because of a work accident
	On the way to work	Whether the patient was on the way to work before visiting the ED
**Vital sign information**		
	Systolic pressure	Systolic blood pressure
	Diastolic pressure	Diastolic blood pressure
	Pulse	Pulse
	Oxygen	Oxygen saturation
	Respiration	Respiration
	Body temperature	Body temperature
	Acute change	Any acute changes before entering the ED
	Fever	Whether the patient has fever
	Pain index	Self-evaluated pain score
	GCS-E	Glasgow Coma Scale score of the patient (eye opening)
	GCS-V	Glasgow Coma Scale score of the patient (verbal response)
	GCS-M	Glasgow Coma Scale score of the patient (motor response)
	Major disease	Whether the patient has an IC^b^ card for severe illness
	Admission count	The number of times the patient went to the hospital in 1 year
	Judgement code	The judgement code for describing the patient’s condition
**Textual data**		
	Chief complaint	The patient’s description of the symptoms
	Judgment description	The record that describes the patient’s symptoms written by the nursing staff

^a^ED: emergency department.

^b^IC: integrated circuit.

### Data Augmentation

After analyzing our prospective data set, we observed an imbalanced data distribution. As machine learning algorithms tend to increase accuracy by reducing errors, most of them are biased toward the majority class and tend to ignore the minority class. For instance, 758 out of 901 (84.1%) ED patients were discharged from the hospital in our prospective data set, and the system could achieve 85% accuracy if it kept on predicting discharge. However, we did not want the system to only indicate discharge. Therefore, to avoid the above situation, we used the “synthetic minority oversampling technique” (SMOTE) to generate some synthesized data to ensure that the system could learn the different patterns between each class. In our study, the iteration of the SMOTE algorithm started by selecting 1 minority sample and finding its top 5 nearest neighbors. These 5 neighbors were chosen to generate new synthesized data by the interpolation method. Finally, the iteration was repeated several times until we obtained the minority class where the number was the same as that of the majority class. However, as the synthesized data may be too diverse, some of the data can have negative influences on the model. Therefore, we used the Tomek Links algorithm to remove some ambiguous data that may hurt model performance by pairing samples and removing the pairs with different labels. An example of the augmentation process is shown in Multimedia Appendix 3. In the original data set, we can observe that only 143 patients are admitted. After applying the SMOTE algorithm on our data set, the number of admitted patients increases to 758. We then use the Tomek Links algorithm to remove some samples that are regarded as ambiguous samples by the algorithm. Finally, in this example, a total of 1294 patients are included in our new augmented prospective data set.

As for text data, since the SMOTE algorithm cannot generate text, we set up a mapping relation to add the text feature for each synthesized sample. First, we created a number of lists, each of which stores the chief complaints from data samples sharing the same class label. After these lists and the synthesized data were ready, for each synthesized sample, we randomly selected 1 chief complaint from the list according to its label and added it as a text feature of the synthesized sample.

#### Pretraining of the Vital Sign Encoder

**Figure 4:**
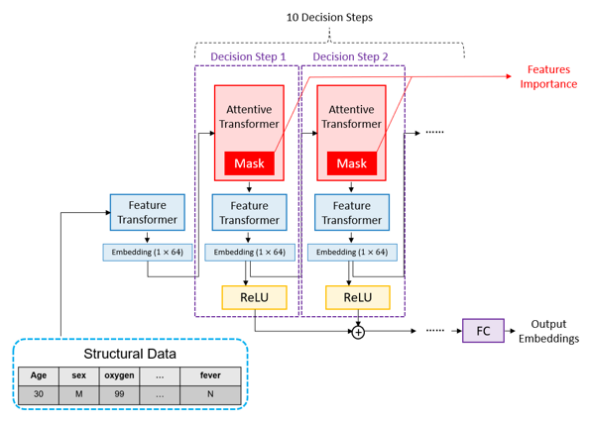
Vital sign encoder architecture (adapted from TabNet). FC: fully connected networks; ReLU: rectified linear unit.

The TabNet architecture is composed of feature transformers and attentive transformers. In TabNet’s design, the mask from the attentive transformer can select the most vital feature from several features, eliminating noise caused by irrelevant features. Furthermore, the mask can be calculated to provide some interpretable information about the feature’s importance. Therefore, considering the objective of this study, our work takes advantage of the encoder-decoder architecture of TabNet, which is inspired by Arik [18], and we adopted this architecture to construct our vital sign encoder (Figure 4).

Before training on the prospective data set, the vital sign encoder was pretrained on retrospective data by unsupervised learning to learn some basic information about such structural data. Structural features of demographics, triage information, and vital sign information (Table 1) were used in this step.

**Figure 5:**
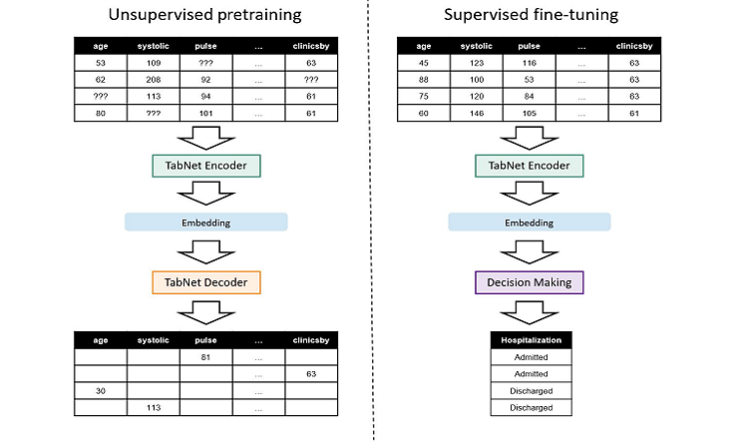
The flow of pretraining the vital sign encoder.

Figure 5 shows the process used for pretraining our vital sign encoder. In triage level prediction and length of stay prediction, since we did not have a triage golden label and length of stay label for pretraining the vital sign encoder, we used only unsupervised learning. On the other hand, both unsupervised learning and supervised learning were used for hospitalization prediction. The reason why we used the unsupervised learning algorithm is that the model can discover hidden data patterns without human intervention by analyzing and clustering the unlabeled information.

In our study, we used the encoder-decoder architecture. We masked some of the input features in our data and then reconstructed the masked features. The reconstruction loss during unsupervised learning is described as follows:



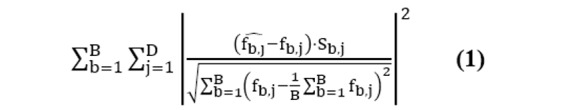



where B denotes the batch size, D denotes the dimension of features (number of features), S is a binary mask sparse matrix with size B×D for masking some of the features, and *f_B_*_×_*_D_* and 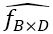 are matrices of features with size B×D representing the ground truth feature values and the predicted outputs, respectively [18].

The purpose of Equation 1 is to calculate the distance between the ground truth feature and the predicted feature. In each iteration, the binary mask *S_b_*_,_*_j_* is sampled independently from Bernoulli distribution, and the mask can only have a value of 0 or 1. During this process involving the masked value and its reconstruction, models are believed to learn implicit relationships between features.

#### Pretraining of the MacBERT Encoder

BERT is a well-known language model that can be used to transform a word into a representation and understand the meaning behind the sentence. In addition, it performs consistently better than other language models (eg, ELMo [12] and GPT [14]) and also performs well in many different tasks. However, although the BERT model can be easily fine-tuned with an additional output layer to achieve outstanding performance, the pretraining process of the model is designed for general purposes. In this study, to better understand our medical data, we repretrained MacBERT (Chinese version of BERT) by applying MLM again. We extracted the text information from the NTUH prospective data set and then used the information to accomplish further training of MLM. All the settings of the training process followed the original set in MacBERT. By further training with MLM, the fine-tuned MacBERT could enrich its knowledge in specific domains.

#### Overall Model Architecture

**Figure 6:**
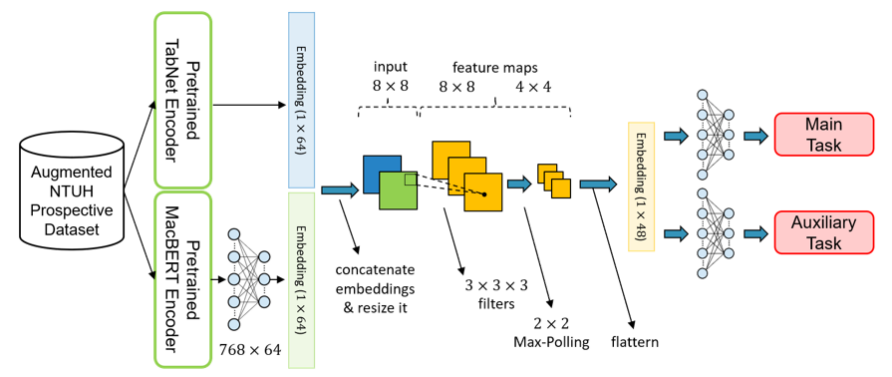
Typical model architecture in the fine-tuning stage. MacBERT: Chinese version of bidirectional encoder representations from transformers; NTUH: National Taiwan University Hospital.

The typical model architecture of each subsystem is shown in Figure 6. After the pretrained encoders are ready, the encoder weights are copied to the fine-tuning stage encoders. The typical model architecture can be divided into 4 main parts: input, encoders, classifiers, and output. First, in the input part, there are 2 data types, namely, structural data and free-text data. Since the prospective data set has only a limited amount of data, we sent it to the augmentation algorithm to obtain synthesized data and added them to the original data set. Second, the structural data and the free-text data are sent to the pretrained TabNet encoder and the pretrained MacBERT encoder, respectively. Afterward, to obtain a comprehensive representation of the data, 2 embeddings coming out from the pretrained TabNet encoder and pretrained MacBERT encoder are concatenated together. Third, the concatenated embeddings are passed through classifiers for output prediction.

#### Input

We used the augmented NTUH prospective data set in the fine-tuning stage. The data set contains 2 data types. The first is structural data, including patient demographics, triage information, and vital sign information. The second is free-text data, including patient chief complaints, nursing staff judgment descriptions, and transformed information from the structural data (Multimedia Appendix 4). However, since MacBERT is a Chinese BERT model, which is trained on simplified Chinese, we translated our text data from traditional Chinese to simplified Chinese to achieve better performance.

#### Encoders

As shown in Figure 6, since there were 2 types of data to be processed, we used the TabNet encoder and MacBERT encoder to extract feature information from structural data and free-text data, respectively. We then transformed these information pieces into high-dimensional embeddings for further training.

##### Pretrained Vital Sign Encoder

We used the pretrained TabNet encoder as our vital sign encoder. In the pretraining stage, we obtained some basic information of these medical data from the NTUH retrospective data set. As a result, to achieve better starting, the pretrained weights were directly deployed into our vital sign encoder. We stacked up 10 decision steps to build our vital sign encoder, and the dimensions of both the input and output were set to 64. A 1×64 vector was the final context vector.

##### Pretrained Language Model Encoder

**Figure 7:**
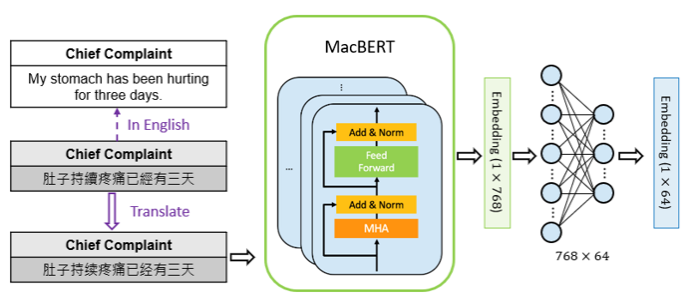
The entire process of handling text data. MacBERT: Chinese version of bidirectional encoder representations from transformers.

As chief complaints are manually recorded by nurses and most of them are written in traditional Chinese, it is better to find a language model that has been trained on a Chinese corpus and can handle Chinese text well. MacBERT is an improved BERT model with novel MLM as a correction pretraining task, which mitigates the discrepancy between pretraining and fine-tuning. Moreover, it has been trained on simplified Chinese corpora, which is more suitable for our work. As a result, we decided to adopt MacBERT from Hugging Face as the chief complaint text encoder in our proposed model, instead of the original BERT model. On the other hand, we observed that the text in our data set might contain different languages, including English and Chinese. Therefore, to make MacBERT applicable to our case, we translated the text into a uniform language, namely, simplified Chinese, before sending it into MacBERT. However, since we wanted the contributions from the vital sign encoder and the MacBERT encoder to be comparable, a fully connected layer was placed after the output vector from MacBERT to decrease the vector dimension from 1×768 to 1×64. The entire process explaining how we handled the text data is shown in Figure 7.

#### Classifiers

**Figure 8:**
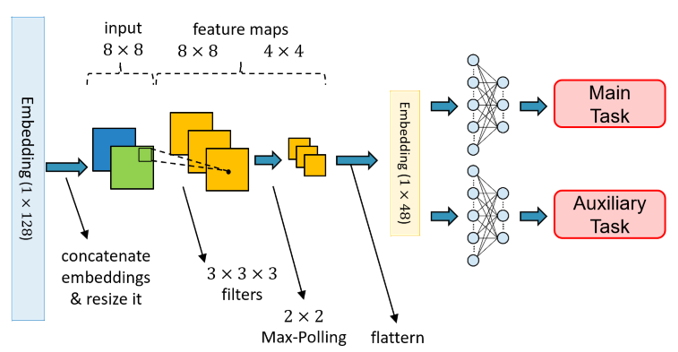
Components of the classifiers.

All the inputs were encoded into high-dimensional embeddings by the encoders mentioned in the previous stage. It is believed that both embeddings have different facets of information; therefore, instead of adding these vectors together, we concatenated these 2 vectors to obtain richer patient information before sending them into the classifiers. Moreover, in our study, we adopted the multi-task learning architecture to learn shared representation and avoid overfitting problems. As a result, there were 2 classifiers for predicting different targets, where each classifier had a 1-layer convolutional neural network and a 2-layer multi-layer perceptron. The details of the process are shown in Figure 8.

#### Output

**Figure 9:**
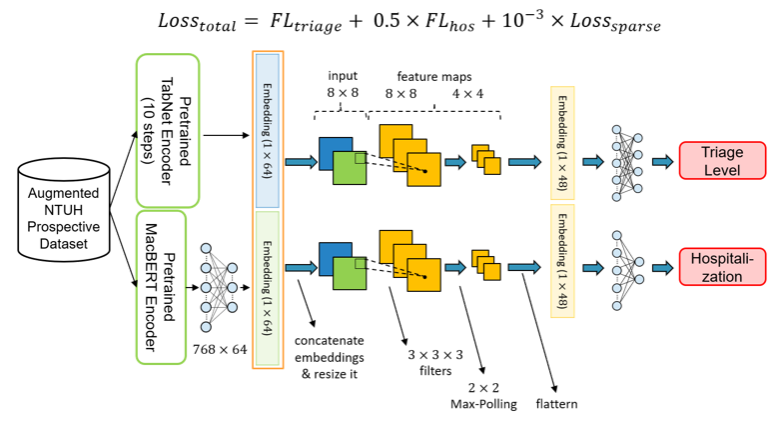
The model architecture of triage level prediction. FL: focal loss; MacBERT: Chinese version of bidirectional encoder representations from transformers; NTUH: National Taiwan University Hospital.

**Figure 10:**
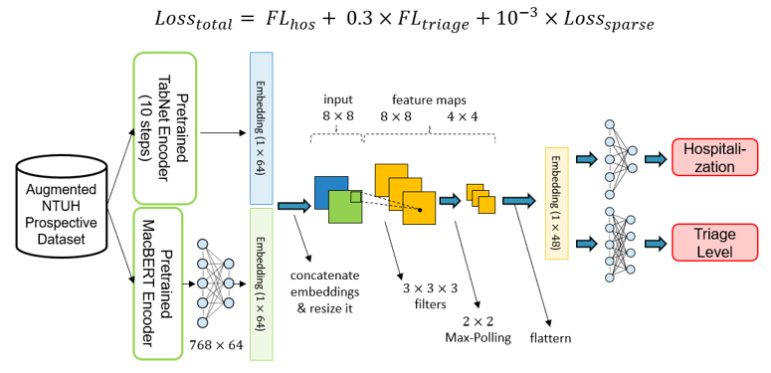
The model architecture of hospitalization prediction. FL: focal loss; MacBERT: Chinese version of bidirectional encoder representations from transformers; NTUH: National Taiwan University Hospital.

**Figure 11:**
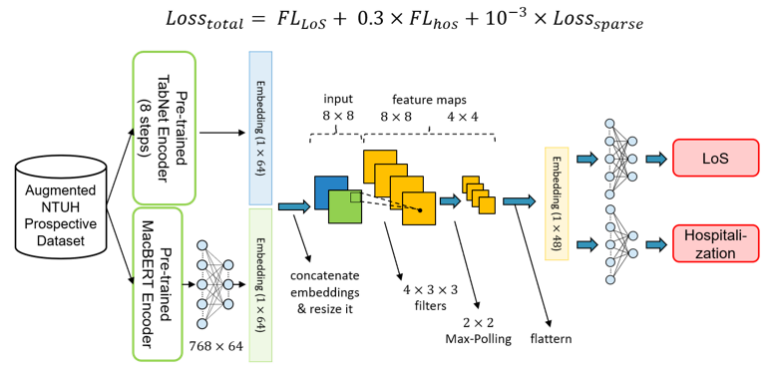
The model architecture of length of stay (LoS) prediction. FL: focal loss; MacBERT: Chinese version of bidirectional encoder representations from transformers; NTUH: National Taiwan University Hospital.

In contrast to most single-output machine learning methods, our proposed model has a multi-task model architecture. Multi-task learning is a type of machine learning method by which the multi-output outcome can be learned simultaneously in a shared model. In addition to the data efficiency advantages, such an approach can reduce overfitting by leveraging auxiliary information and allowing fast learning. Since target prediction loss will update the encoders, the encoders can avoid being overfitted and learn more general knowledge. As there were 3 medical outcomes in our system, we designed 3 models with slight differences to handle different tasks. The details of these 3 models are shown in Figures 9 to 11.

#### Loss Function

Total loss combines focal loss and sparse entropy loss as follows:



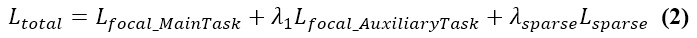



where λ_1_ is a hyperparameter for determining the learning direction of the model via controlling the balance between the main task and related task, and λ_sparse_ is a hyperparameter for controlling the sparsity of the TabNet encoder, where a greater parameter is associated with a greater effect of the tabular data on the entire model, and the TabNet encoder tends to select 1 feature in 1 decision step.

In order to assess the performance of the model, the focal loss function was utilized by comparing the ground truth label with the probability distributions over network predictions, which has been shown as follows:



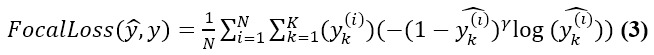



where *ŷ* is the model prediction, *y* is the ground truth value, superscript *i* refers to sample *i*, *y_k_* is 0 or 1 (indicating whether a class label is the correct classification among K classes), 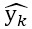 denotes the confidence score of class k, and γ is a hyperparameter that is set to 2 in our study.

TabNet uses sparse entropy loss (first proposed in [23]) to provide a favorable inductive bias for data sets where most features are redundant. The sparse entropy loss can not only help the model to select salient features from all attributes of the sample, but also fasten the training process. The equation is as follows:



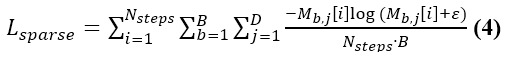



where *N_steps_* denotes how many decision steps are stacked up in the model, B is the batch size, D is the total number of features, M represents the mask, M*_b_*_,_*_j_* [i] refers to the mask at the *i^th^* step with batch sample b and feature j, and ε is a small number to maintain numerical stability.

## Results

### Experimental Setup

A series of experiments were conducted to validate the effectiveness of our design. The details of our system environment are presented below. We conducted our experiments on the Ubuntu 20.04 operating system with PyTorch 1.7.1 and Python 3.9.7, and all training procedures were performed on a computer with a Nvidia RTX 3090 graphics card, an Intel Core i7-1070K processor, and 32 GB of RAM.

### Training Settings

The Adam optimizer with an initial learning rate of 0.01 was used in our experiments, and it was adjusted by the “ReduceLROnPlateau” scheduler with the patient value set as 15. Meanwhile, if the loss did not improve for 50 epochs, an early stop action was taken.

All experiments were carefully conducted in the following steps: (1) The data set was divided into 3 parts (training set, validation set, and testing set in the ratio of 8:1:1); (2) The training set was used to generate synthesized data to make up the gap between classes, and the synthesized data were added into the original training data set; (3) Our design was evaluated by taking the average test performance for 10 trials, as the division of the data set might have varied effects on the experiment results.

### Evaluation Metrics

Since our data set was obviously imbalanced, the accuracy performance cannot represent the effectiveness of our system. As a result, in our experiment, the evaluation metrics included precision, recall, and F1-score. Precision measures the rate of ground truth classes that are predicted correctly. Recall measures the portion of each class of our prediction that is actually that class. Finally, F1-score represents the harmonic mean between precision and recall. Their formulas are as follows:



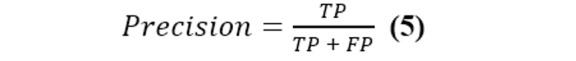





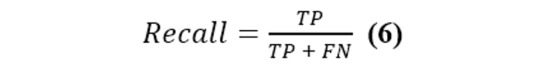





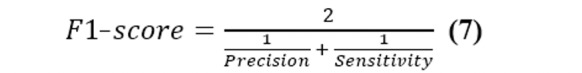



where TP, TN, FP, and FN denote true positive, true negative, false positive, and false negative, respectively.

### Data Characteristics

Our study included 2 data sets. One data set was the NTUH retrospective data set, which contains a collection of the past EHRs of 268,716 visits from 2009 to 2015, and the other data set was the NTUH prospective data set, which contains data collected with patient consent in the NTUH ED from May 26, 2020, to February 21, 2022, and includes 901 ED patient records after removal of unreasonable and missing data. Table 2 summarizes the data characteristics of vital sign information in these 2 data sets. Despite similar average values across all fields in the 2 data sets, on performing statistical tests using *P*-values, we found that there was a significant difference between the 2 data sets. However, we believe that using data with the same data collection background but different distributions can still effectively improve the robustness and generalization ability of the model. By pretraining on diverse data, the model can learn more general representations, leading to improvements in the final predictions.

On the other hand, the distributions for different tasks are shown in Multimedia Appendix 5. It is worth mentioning that the distribution gap of the triage level between the retrospective data set and prospective data set was greater than the distribution gaps for hospitalization and length of stay. This is because hospitalization and length of stay are based on facts, and in contrast to the triage level in the retrospective data set, the triage level in the prospective data set comes from physician diagnosis. As it is believed that the doctor’s triage level can assign patient acuity more accurately, we used it as our golden label for predicting the triage level. Another reason for the distribution gap could be the difficulty in collecting data from more severe patients.

**Table 2:** Patient characteristics in the National Taiwan University Hospital retrospective and prospective data sets.

Variable		NTUH^a^ retrospective data set	NTUH prospective data set
Age (years), mean (SD)		49.1 (19.98)	52.4 (18.98)
**Sex, n (%)**			
	Female	141,783 (52.8)	450 (50.1)
	Male	126,933 (47.2)	450 (49.9)
**Arrival time, n (%)**			
	7 AM to 3 PM	10,2256 (42.8)	518 (57.4)
	3 PM to 11 PM	11,4970 (38.0)	289 (32.1)
	11 PM to 7 AM	5,1490 (19.2)	94 (10.5)
Systolic blood pressure (mmHg), mean (SD)		136.3 (26.79)	132.4 (24.78)
Diastolic blood pressure (mmHg), mean (SD)		80.8 (15.22)	79.8 (13.91)
Pulse (beats/min), mean (SD)		88.8 (18.74)	89.5 (18.74)
Oxygen saturation (%), mean (SD)		97.0 (3.09)	97.7 (1.69)
Respiration (breaths/min), mean (SD)		18.2 (2.16)	18.8 (2.04)
Body temperature (°C), mean (SD)		37.0 (0.82)	36.7 (0.65)
**Pain index (scale), n**			
	0	134,292	357
	1-3	9,554	368
	4-6	60,526	140
	7-10	64,344	36

^a^NTUH: National Taiwan University Hospital.

### Experimental Results

We compared our model’s performance regarding triage level, hospitalization, and length of stay against the performance of other machine learning methods. As the data of only 901 ED visits were finally included in our study, it was a challenge to obtain a robust model with great capability to identify critical patients.

Unlike other work on triage level prediction, since we endeavored to fix the bias of traditional rule-based system triage, such as the ESI and TTAS, we used the diagnosis results provided by the physician as our golden label. As shown in Table 3, it is worth noting that our triage model achieved a nearly 30% improvement in 4 metrics, including accuracy, precision, recall, and F1-score, when compared to the results obtained from other models. These outstanding results show the promising potential of our proposed model.

As shown in Table 4, we can observe that our hospitalization model achieved the highest performance in 3 metrics, including precision, recall, and F1-score. Although the support vector machine (SVM) model achieved an accuracy of 91.2%, it may tend to predict the majority (discharge) owing to the low precision and recall values. From the previous discussion, it can be seen that our model is the most discriminative model.

Additionally, our proposed model outperformed other models. Although the study design and data set in our study are different from those in other studies, it is worth indicating that with the help of retrospective data pretraining, the model can learn more than with only the use of prospective data. Our proposed model achieved promising results, with 3%-6% improvement in accuracy (Table 5).

As shown in Table 6, although most of the models achieved an accuracy of higher than 70%, their performances on other metrics revealed that these models tend to predict the majority class. Nevertheless, except for accuracy, our length of stay model outperformed other machine learning methods in the other 3 metrics, indicating the capability of our length of stay model for discrimination.

**Table 3:** Performance comparison between our model and other machine learning methods in the “triage level” task.

Method	Accuracy	Precision	Recall	F1-score
TabNet [18]	0.425	0.436	0.410	0.423
NODE^a^ [19]	0.472	0.324	0.328	0.324
Random forest [24]	0.354	0.506	0.300	0.376
XGBoost^b^ [15]	0.351	0.394	0.308	0.345
SVM^c^ [25]	0.340	0.581	0.268	0.367
Our model	0.633^d^	0.686^d^	0.633^d^	0.658^d^

^a^NODE: neural oblivious decision ensembles.

^b^XGBoost: extreme gradient boosting.

^c^SVM: support vector machine.

^d^Highest value.

**Table 4:** Performance comparison between our model and other machine learning methods in the “hospitalization” task.

Methods	Accuracy	Precision	Recall	F1-score
TabNet [18]	0.791	0.701	0.702	0.701
NODE^a^ [19]	0.752	0.622	0.689	0.653
Random forest [24]	0.821	0.765	0.674	0.717
XGBoost^b^ [15]	0.829	0.651	0.679	0.655
SVM^c^ [25]	0.912^d^	0.456	0.500	0.477
Our model	0.822	0.811^d^	0.823^d^	0.817^d^

^a^NODE: neural oblivious decision ensembles.

^b^XGBoost: extreme gradient boosting.

^c^SVM: support vector machine.

^d^Highest value.

**Table 5:** Performance comparison between our model and the models in other related studies in the “hospitalization” task.

Study	Data set	Study type	Accuracy	Precision	Recall	F1-score
Study by Raita et al [26]	NHAMCS^a^	Retrospective	—^b^	—	0.750	—
Study by Yao et al [27]	NHAMCS	Retrospective	0.775	0.820^c^	0.790	0.804
Study by Leung et al [28]	NTUH^d^	Prospective	0.805	0.806	0.790	0.798
Our study	NTUH	Prospective	0.822^c^	0.811	0.823^c^	0.817^c^

^a^NHAMCS: National Hospital Ambulatory Medical Care Survey.

^b^Not reported.

^c^Highest value.

^d^NTUH: National Taiwan University Hospital.

**Table 6:** Performance comparison between our model and other machine learning methods in the “length of stay” task.

Methods	Accuracy	Precision	Recall	F1-score
TabNet [18]	0.683	0.654	0.665	0.659
NODE^a^ [19]	0.721	0.616	0.589	0.602
Random forest [24]	0.754	0.606	0.444	0.512
XGBoost^b^ [15]	0.744	0.523	0.446	0.481
SVM^c^ [25]	0.791^d^	0.263	0.333	0.294
Our model	0.713	0.786^d^	0.713^d^	0.747^d^

^a^NODE: neural oblivious decision ensembles.

^b^XGBoost: extreme gradient boosting.

^c^SVM: support vector machine.

^d^Highest value.

### Ablation Studies

#### Effectiveness of Multimodality

Experiments were conducted to demonstrate the superior performance of our proposed model. Since our model comprised the TabNet encoder and the language model encoder, we designed an experiment to show that the performance of a model leveraging both vital sign information and text information is better than that of a model using only 1 information modality. Table 7 shows that the proposed model achieved the best performance when both modalities were used. The results suggest that both structural and text data contribute to model prediction. The greater performance of the model using only tabular data than that using only text data could be attributed to the advantage of pretraining, as the vital sign encoder was pretrained with a large volume of retrospective data.

**Table 7:** The effectiveness of different modalities in the “triage level” task.

Methods	Accuracy	Precision	Recall	F1-score
Only tabular data	0.575	0.613	0.568	0.589
Only text data	0.439	0.119	0.250	0.162
Our method (tabular data + text data)	0.633^a^	0.686^a^	0.633^a^	0.658^a^

^a^Highest value.

#### Effectiveness of Multitask Training and Data Augmentation

Multitask learning experiments confirmed that the approach does offer advantages like improving data efficiency, reducing overfitting through shared representations, and allowing fast learning by leveraging auxiliary information. However, in order to obtain a more robust feature extractor, in a general setting, the targets in the multitask learning model should be related. As a result, in the experiments, we selected triage level prediction and hospitalization as our 2 outputs. It is believed that a patient assigned to level 1 or 2 should have a higher probability of admission to the hospital after being discharged from the ED. Moreover, since data distribution in triage labels is unbalanced, we attempted to narrow the distribution gap by using the method of data augmentation. Table 8 shows that both multitask learning and augmentation contributed to better performance.

**Table 8:** The effectiveness of different architectures in the “triage level” task.

Methods	Accuracy	Precision	Recall	F1-score
Multitask	0.500	0.369	0.500	0.425
Single task + augmentation	0.583	0.600	0.582	0.591
Single task	0.458	0.506	0.455	0.479
Our method (multitask + augmentation^a^)	0.633^b^	0.686^b^	0.633^b^	0.658^b^

^a^The method of data augmentation used in our proposed model is described in the “Data Augmentation” subsection.

^b^Highest value.

#### Effectiveness of Different Language Models

Experiments were conducted to evaluate the performance between different language models (Table 9). In our original data set, the chief complaint was written in traditional Chinese. However, no language model has been trained on traditional Chinese. Hence, to solve this problem, we first translated the text features into different languages before sending them to the respective language models. The results showed that the model using MacBERT as the language encoder was better than models using other approaches.

**Table 9:** The effectiveness of different language models in the “triage level” task.

Methods	Data language	Accuracy	Precision	Recall	F1-score
Multilingual BERT^a^	Simplified Chinese	0.500	0.369	0.500	0.425
Multilingual BERT	English	0.583	0.600	0.582	0.591
BERT	English	0.458	0.506	0.455	0.479
Our method (MacBERT^b^)	Simplified Chinese	0.633^c^	0.686^c^	0.633^c^	0.658^c^

^a^BERT: bidirectional encoder representations from transformers.

^b^MacBERT: Chinese version of BERT.

^c^Highest value.

### Effectiveness of Different Fusion Methods

Experiments were conducted to demonstrate the superior performance of our proposed model. As our model directly concatenated the decreased embedding from the language model and the embedding from the vital sign encoder, we designed an experiment to show that it is necessary to make contributions for the text data and structural data to be comparable, and direct concatenation fusion can preserve more information than addition fusion. In Table 10, the first experiment involves the model adding 2 embeddings (text and vital sign embeddings) together with a learnable scale value to balance the gap between the text and vital sign embeddings, and the second experiment involves directly using the embedding from the language model instead of passing another fully connected network to decrease its dimension. The results suggest that making 2 embeddings to be comparable and using a direct concatenation fusion method can contribute to better performance.

**Table 10:** The effectiveness of different fusion methods in the “triage level” task.

Methods	Accuracy	Precision	Recall	F1-score
Experiment 1 (addition fusion)	0.548	0.580	0.547	0.563
Experiment 2 (no concatenation fusion)	0.583	0.634	0.583	0.607
Our method	0.633^a^	0.686^a^	0.633^a^	0.658^a^

^a^Highest value.

### Interpretability

Although machine learning models can provide remarkably good prediction results, models need to provide explanations of the results that humans can understand easily. In our proposed model, for structural features, the attentive transformer from TabNet generated the mask to mask out different features in each decision step and observed how these features affect the model performance. As a final step, the attentive transformer calculated the importance of features by adding up the mask values of each step. On the other hand, BertViz [29] is an interactive tool that can visualize attention in transformer language models such as BERT. By acquiring attention scores from transformer layers in language models, BertViz can point out important words that contribute to the predicted result.

Multimedia Appendix 6 provides an inference example from the field test, and Multimedia Appendix 7 provides the prediction results of the inference sample for hospitalization. In this example, the patient shows acute change during the triage process, extremely high systolic and diastolic blood pressure, and an unusual Glasgow Coma Scale (GCS) score. As shown in Multimedia Appendix 7 our system recommended admission of the inferenced patient, and the patient was actually admitted to the hospital. Our system not only successfully provided the correct suggestion to the nursing staff, but also indicated that acute change, systolic blood pressure, diastolic blood pressure, GCS-E, and GCS-M have important effects on the prediction result. As for text analysis, we used the concept from BertViz to extract attention scores for each token from the language model and visualize these attention scores. Although the language model had a hierarchy of linguistic signals from phrase to semantic features, it is believed that the deeper layer of the language model holds more information of the whole sentence [30]. Hence, we extracted the attention score from the ninth layer of the language model for further visualization (Multimedia Appendix 8).

### System Application

Triage aims to prioritize patients in the ED and ration care toward those patients who need immediate care. However, recently, owing to the rising number of elderly patients and the high volume of low-acuity ED visits under waiting, patients tend to wait for very long to see the physician. This situation can cause several severe clinical outcomes such as increased mortality rates.

With the advancement in technology and popular application of computers nowadays, we wonder whether machine learning methods can help to mitigate the overcrowding problem in the ED. Therefore, we developed a triage system based on our proposed model and adopted it in the NTUH ED to provide stable and reasonable clinical AI suggestions to nursing staff. For application in the real world, we should take the running time of the system into account. The entire running time of each part is shown in Multimedia Appendix 9. The system takes no more than 10 seconds to make clinical predictions.

Before the system is officially launched, we planned a field test to ensure that the system can achieve promising performance in the real world. Finally, we included almost 6500 ED patients in our analysis from September 30, 2022, to December 30, 2022. The distributions of hospitalization and length of stay between these patients were quite different as compared to the NTUH prospective data set (Multimedia Appendix 10 and Multimedia Appendix 11). Especially for length of stay, patients who stayed in the ED for over 24 hours were much less in this data set than in both NTUH data sets (Multimedia Appendix 11). Moreover, since our golden triage level depended on the physician’s diagnosis, it was challenging to label all patients in the field test; however, we evaluated our system in another way, which will be discussed later. The distribution gap between both NTUH data sets and the field test is presented in Multimedia Appendix 12.

As shown in Multimedia Appendix 13 and Multimedia Appendix 14, there was a slight performance gap between the experiments on the earlier mentioned data sets and the real-world data. However, from the results of the confusion matrix, it can be seen that in the case of “patients actually discharged,” 2085 out of 2539 (82.1%) discharged patients were accurately predicted and were recommended to be discharged by the system. On the other hand, in the case of “patients actually admitted,” 194 out of 316 (61.4%) patients were accurately predicted and were recommended to stay in the hospital.

As mentioned previously, for length of stay, there was a large distribution gap between our field test data set and the NTUH prospective data set. Multimedia Appendix 15 and Multimedia Appendix 16 show that the system cannot perform as good as it does in local experiments. However, from the results of the confusion matrix, we can observe that the system has a better capability of discriminating patients who stay for less than 6 hours, and the system tends to underestimate patients who stay in the ED for 6 to 24 hours.

Finally, Multimedia Appendix 17 and Multimedia Appendix 18 show that although the newly collected data did not have the golden triage level labels provided by the doctors, the distribution of the triage level indicated that the model predicted a fairly even distribution, while the system triage still mainly predicted level 3.

## Discussion

### Limitations

Although our proposed model showed good preliminary results compared to the results of other machine learning methods, it still has a long way to go. For instance, despite our model’s ability to incorporate various language models, it may not perform well for languages where specific language models are not available in the training data set. Second, as we need to translate the text into a uniform language initially and the sentence in the data is not always complete, a better translator and some postprocessing techniques are needed to alleviate the problems. Additionally, as retrospective data lack a label in triage level prediction, expansion of the data set for training the model should help the model to learn a wider range of patterns and should enhance model performance. Moreover, since our proposed model can allow efficient learning of image or text encoders in the presence of multimodality along with tabular data, further work can add images or speech information into our model to help it achieve better performance.

### Conclusion

Emergency services are an essential aspect of the health care system in hospitals, and the demand for these services has increased exponentially in recent years. Although Taiwan has established a standard process of assigning patients to different emergency levels, there is insufficient capacity to ensure precise assignment. Most patients are over-triaged or under-triaged, which can waste limited medical resources or have severe consequences such as patient mortality.

In this study, we aimed to design a deep learning prediction system that can prioritize patients and assign patients to appropriate triage levels. To obtain rich information from patients, our proposed model not only uses vital sign information, but also leverages text information.

Our system included a well-pretrained vital sign encoder and a repretrained MacBERT encoder. Additionally, by using the multitask learning and data augmentation method, we successfully obtained promising results for triage level prediction, hospitalization prediction, and length of stay prediction. For triage level prediction, there were nearly 30% improvements in 4 metrics compared with other machine learning methods, including accuracy, precision, recall, and F1-score. Different modalities and model architectures have also been studied for ablation effectiveness. Moreover, our proposed model also provides clinicians with interpretability to understand the reasons behind the model predictions.

In conclusion, our system improved the prediction of 3 different medical outcomes when compared with other machine learning methods. With the pretrained vital sign encoder and repretrained MLM MacBERT encoder, our multimodality model can provide a deeper insight into the characteristics of EHRs. Additionally, by providing interpretability, we believe that the proposed system can assist nursing staff and physicians in taking appropriate medical decisions.

## Appendix

Multimedia Appendix 1 Comparison between related studies and our proposed work.

Multimedia Appendix 2 An example of uncleaned data.

Multimedia Appendix 3 An example of the number change in the data using the synthetic minority oversampling technique (SMOTE) algorithm and Tomek Links algorithm.

Multimedia Appendix 4 The data construction of the input (left is the structural data; right is the free-text data).

Multimedia Appendix 5 Distribution of each task.

Multimedia Appendix 6 An inference example from the field test.

Multimedia Appendix 7 Prediction result and feature importance of the inferenced patient for hospitalization from the field test (structural data).

Multimedia Appendix 8 Text visualization of the inference patient for hospitalization from the field test.

Multimedia Appendix 9 The running time of each part in the system.

Multimedia Appendix 10 The distribution gap between both National Taiwan University Hospital data sets and the field test for hospitalization.

Multimedia Appendix 11 The distribution gap between both National Taiwan University Hospital data sets and the field test for length of stay.

Multimedia Appendix 12 The distribution gap between both National Taiwan University Hospital data sets and the field test.

Multimedia Appendix 13 The performance of hospitalization in the field test.

Multimedia Appendix 14 The performance (truth) of hospitalization in the field test.

Multimedia Appendix 15 The performance of length of stay in the field test.

Multimedia Appendix 16 The performance (truth) of length of stay in the field test.

Multimedia Appendix 17 The prediction of triage in the field test.

Multimedia Appendix 18 Graph showing the prediction of triage in the field test.

## Abbreviations

AI: artificial intelligence
BERT: bidirectional encoder representations from transformers
CatBoost: category boosting
C-NLP: clinical natural language processing
DT: decision tree
ED: emergency department
EHR: electronic health record
ELMo: embeddings from language models
ESI: Emergency Severity Index
GCS: Glasgow Coma Scale
GPT: generative pretrained transformer
MacBERT: Chinese version of bidirectional encoder representations from transformers
MLM: mask language modeling
NLP: natural language processing
NTUH: National Taiwan University Hospital
SMOTE: synthetic minority oversampling technique
TTAS: Taiwan Triage Acuity Scale
XGBoost: extreme gradient boosting
