# Supplementary figures and images for: Interpretable Deep Learning System for Identifying Critical Patients Through the Prediction of Triage Level, Hospitalization, and Length of Stay: Prospective Study
Source: JMIR Med Inform. 2024 Apr 1;12:e48862. doi: 10.2196/48862 (PMC11019422; doi:10.2196/48862)

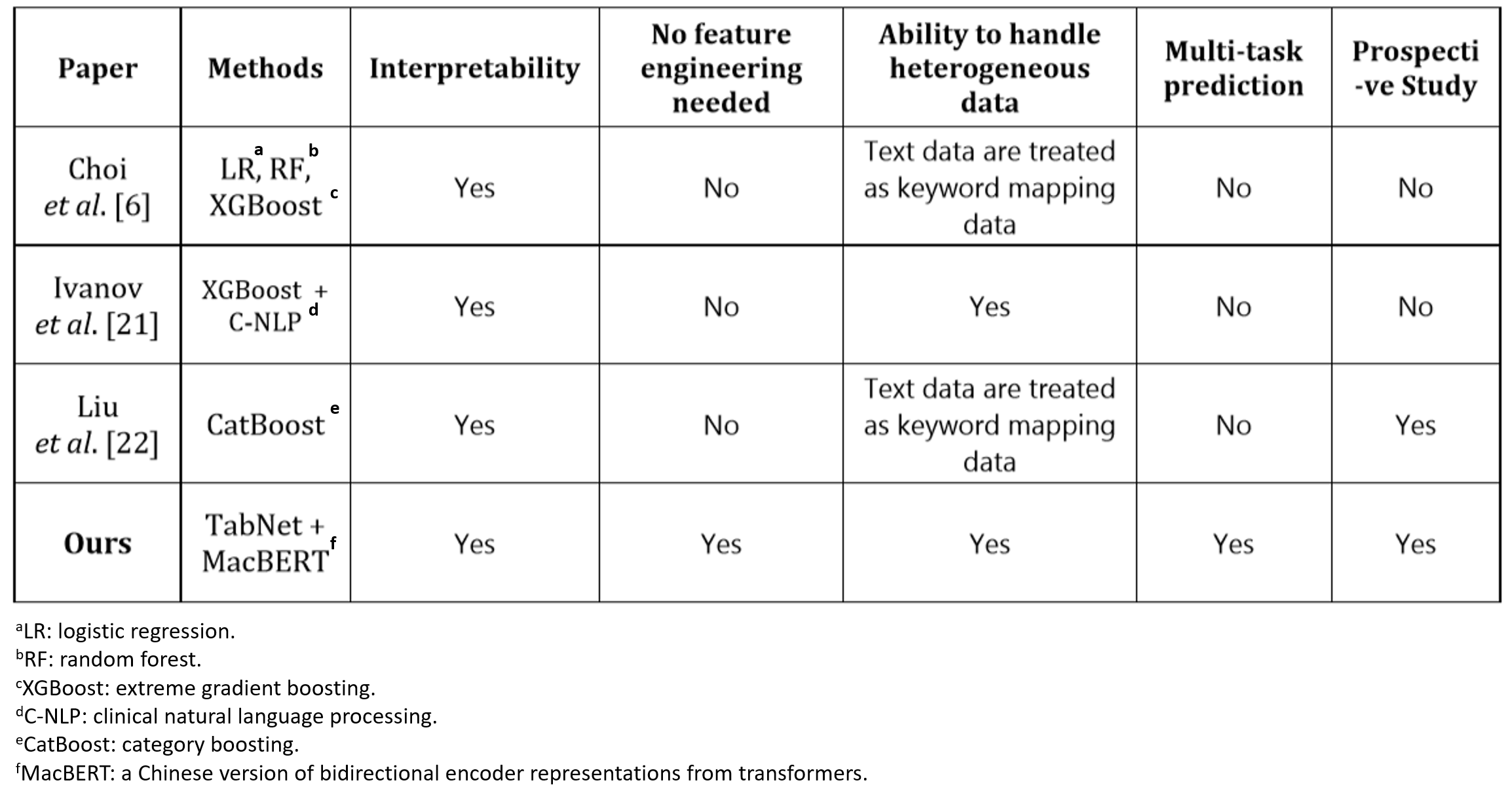

Supplement: Multimedia Appendix 1 [file medinform_v12i1e48862_app1.png]

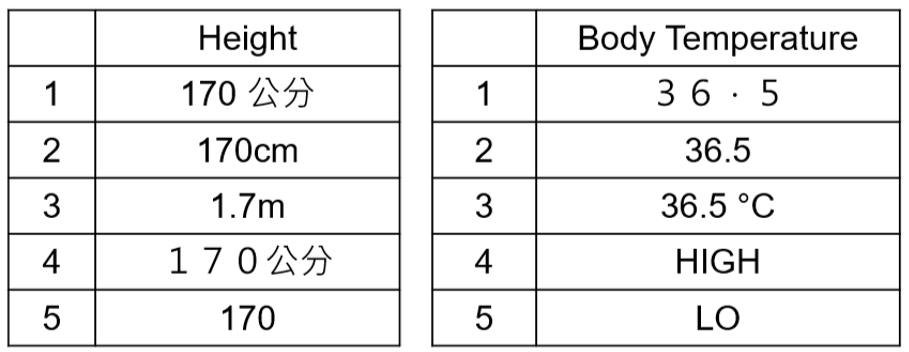

Supplement: Multimedia Appendix 2 [file medinform_v12i1e48862_app2.png]

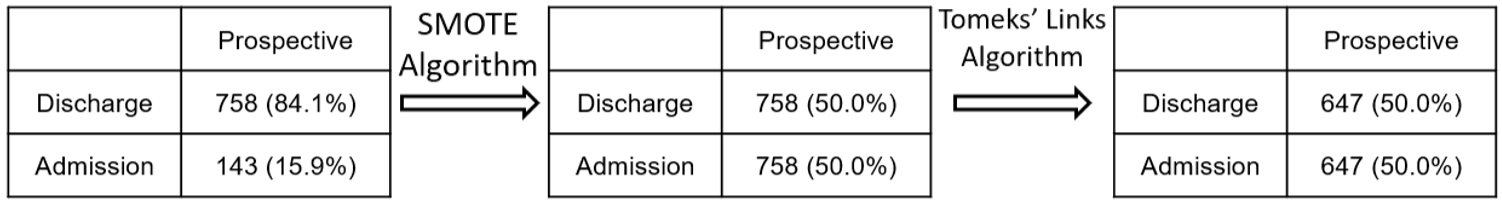

Supplement: Multimedia Appendix 3 [file medinform_v12i1e48862_app3.png]

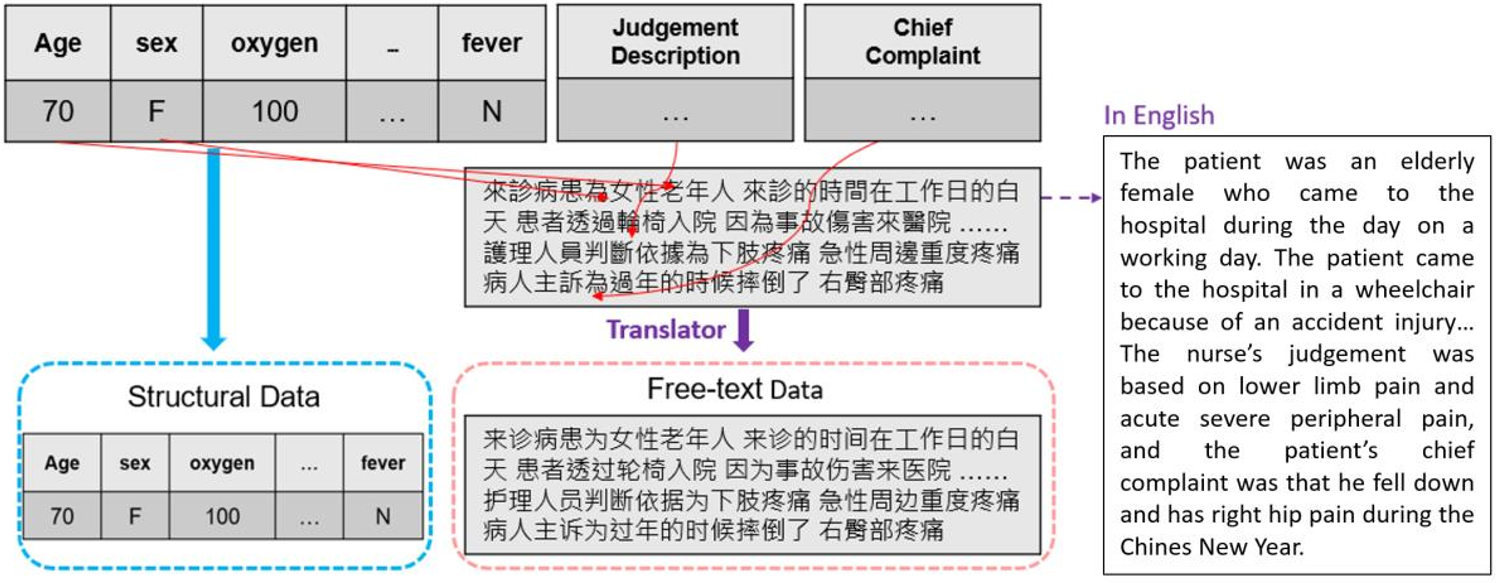

Supplement: Multimedia Appendix 4 [file medinform_v12i1e48862_app4.png]

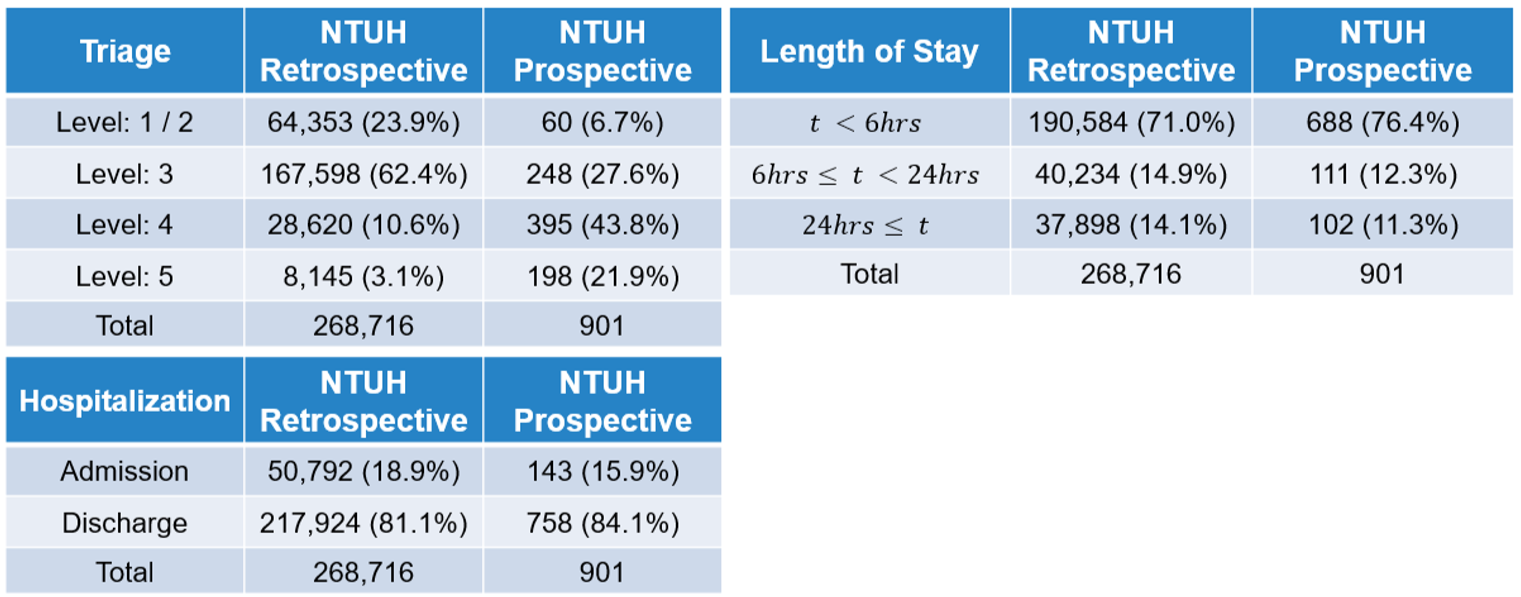

Supplement: Multimedia Appendix 5 [file medinform_v12i1e48862_app5.png]

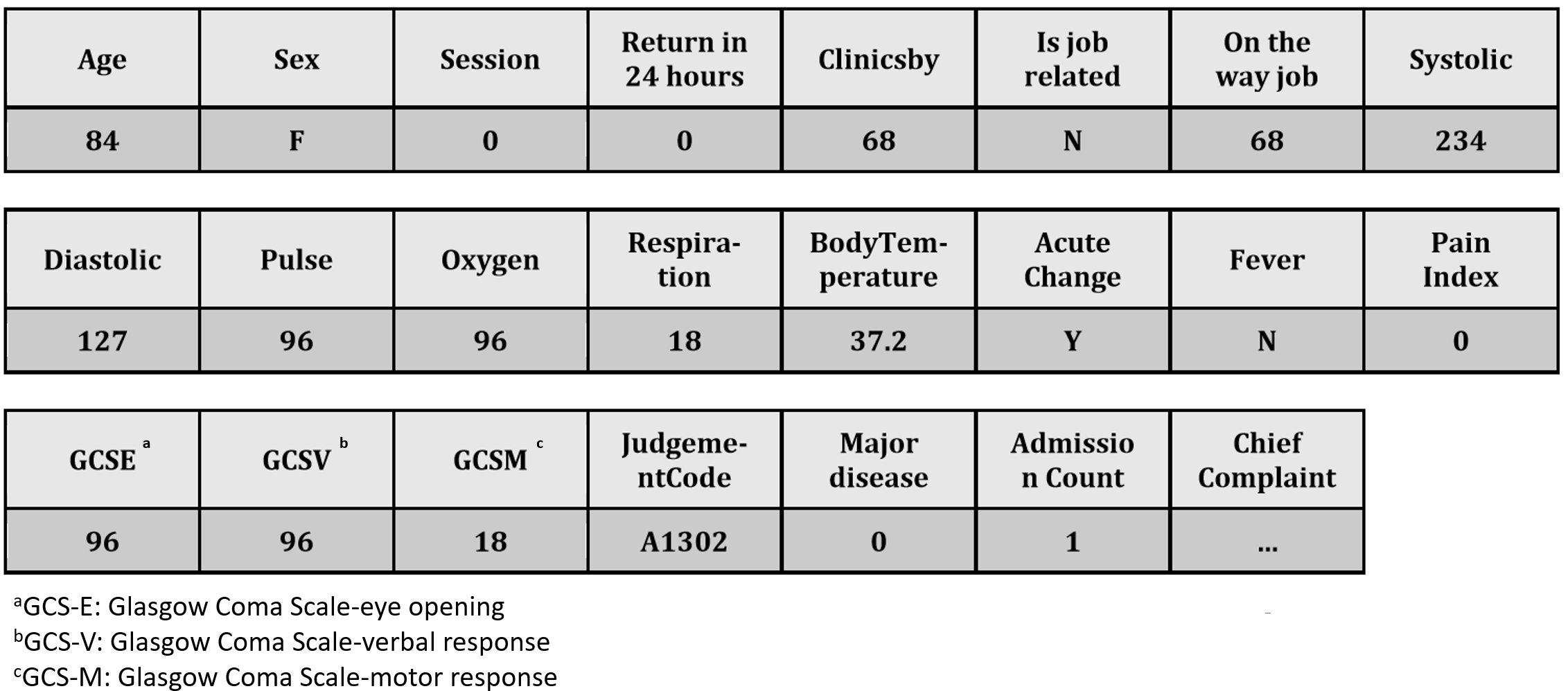

Supplement: Multimedia Appendix 6 [file medinform_v12i1e48862_app6.png]

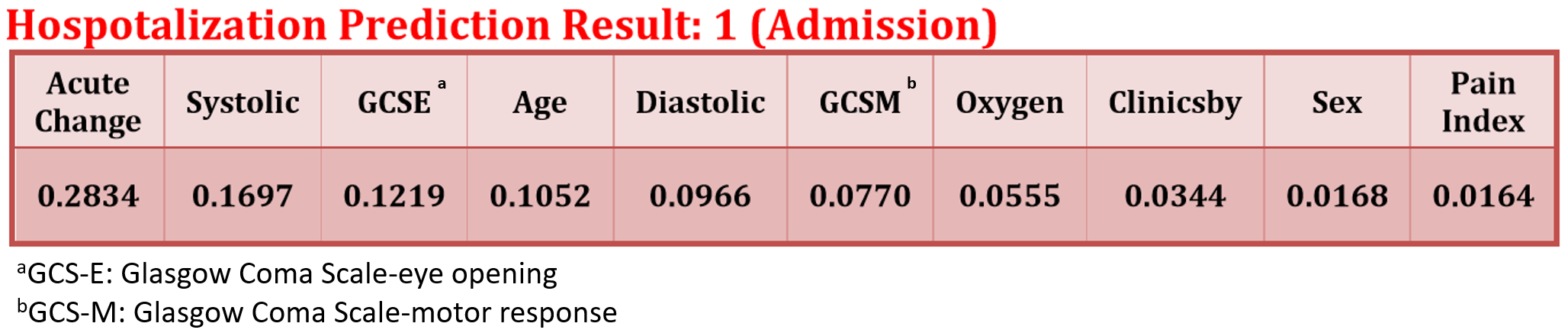

Supplement: Multimedia Appendix 7 [file medinform_v12i1e48862_app7.png]

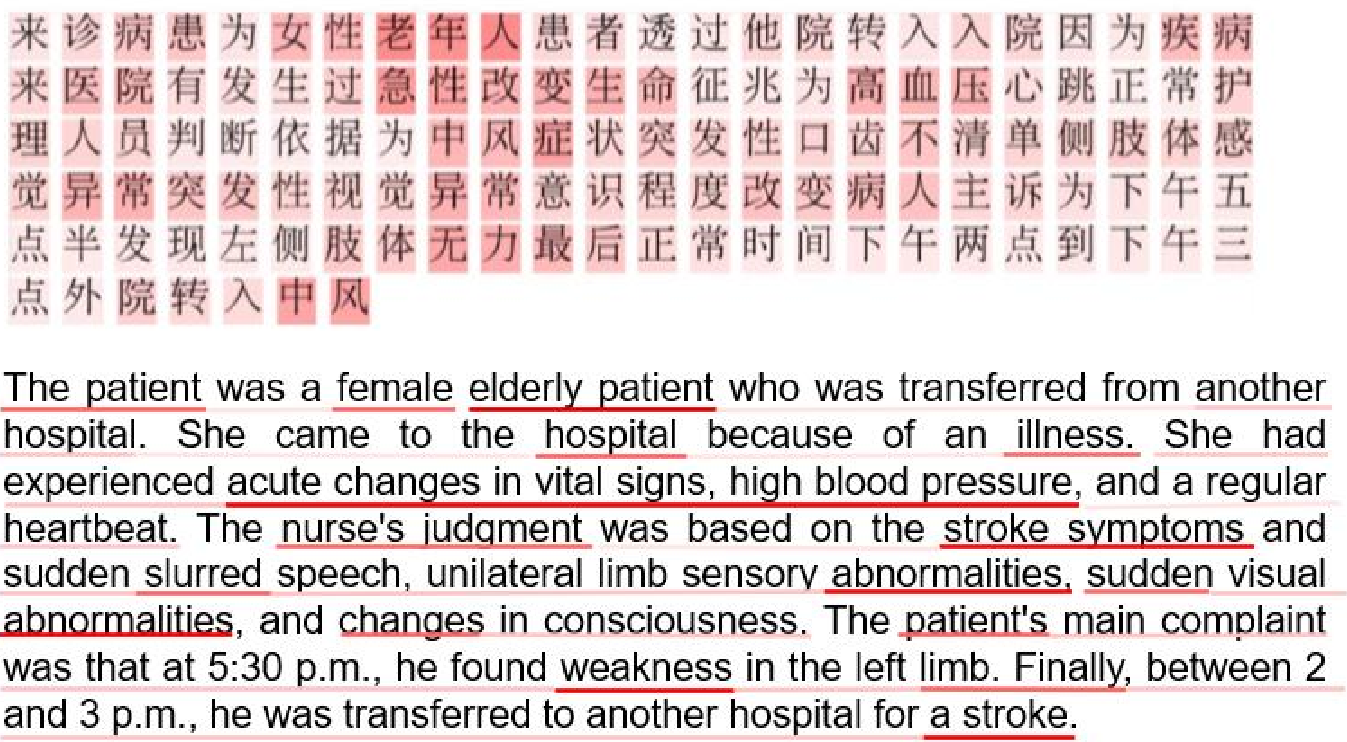

Supplement: Multimedia Appendix 8 [file medinform_v12i1e48862_app8.png]

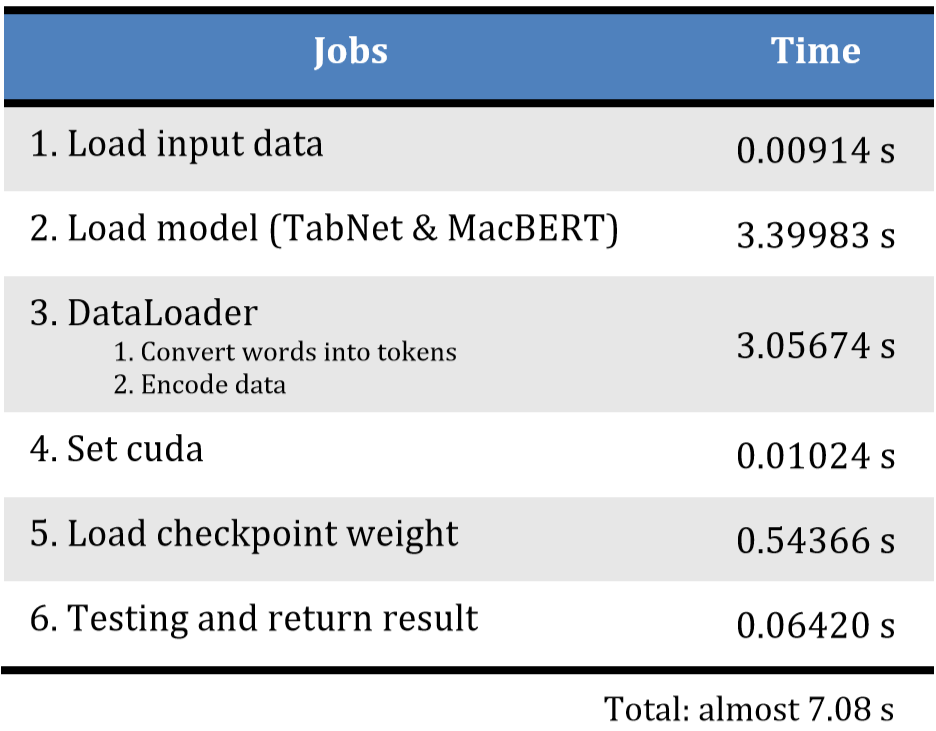

Supplement: Multimedia Appendix 9 [file medinform_v12i1e48862_app9.png]

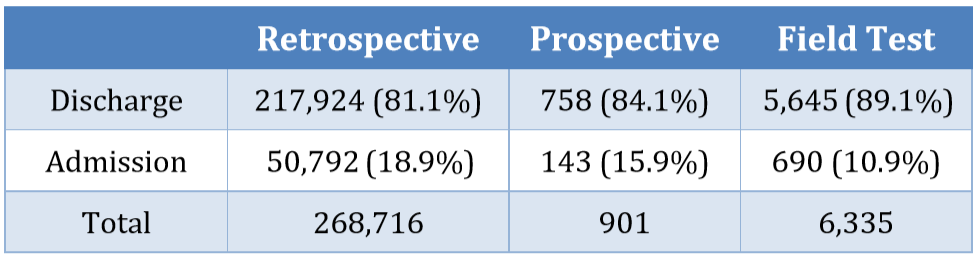

Supplement: Multimedia Appendix 10 [file medinform_v12i1e48862_app10.png]

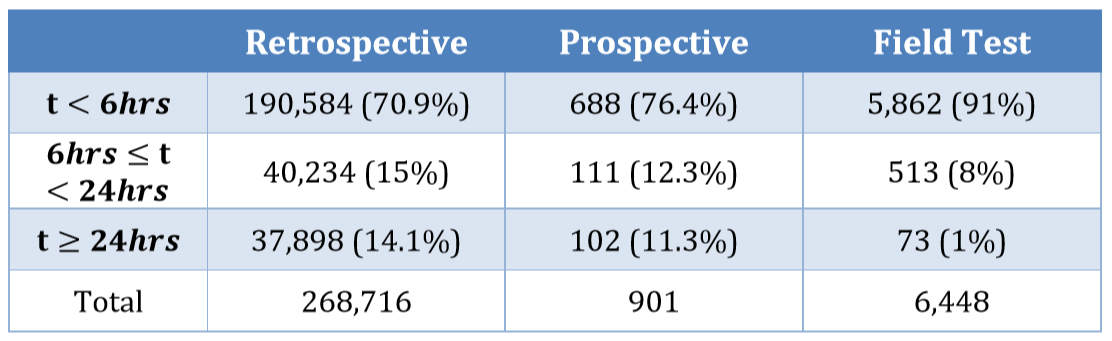

Supplement: Multimedia Appendix 11 [file medinform_v12i1e48862_app11.png]

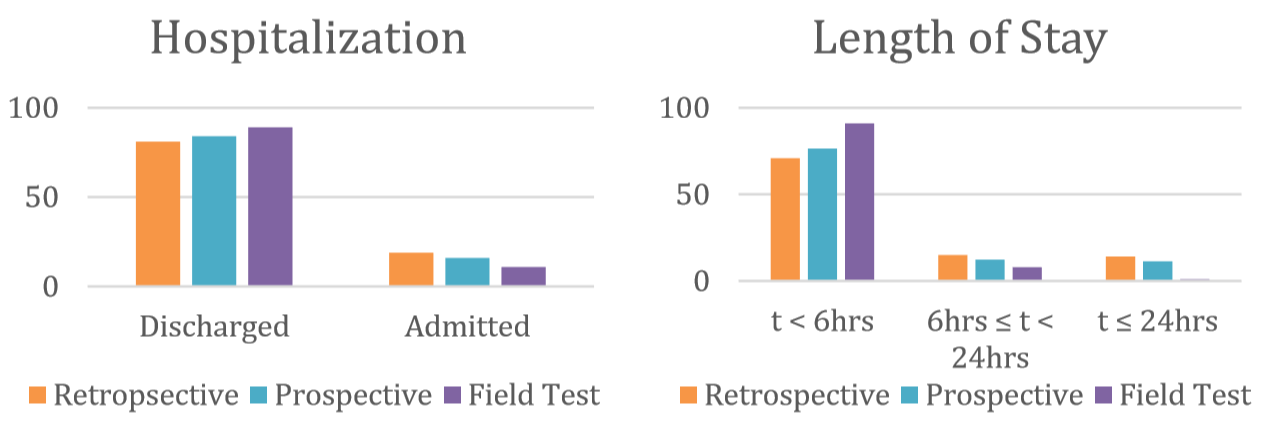

Supplement: Multimedia Appendix 12 [file medinform_v12i1e48862_app12.png]

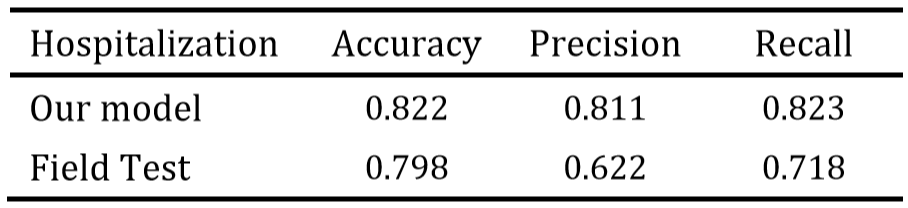

Supplement: Multimedia Appendix 13 [file medinform_v12i1e48862_app13.png]

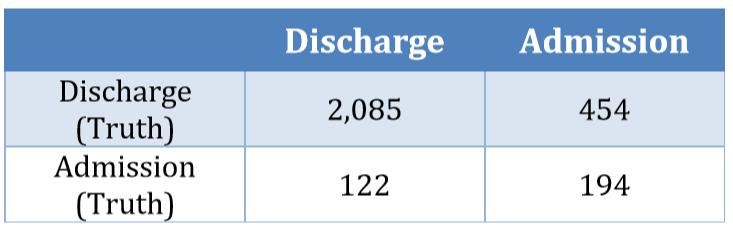

Supplement: Multimedia Appendix 14 [file medinform_v12i1e48862_app14.png]

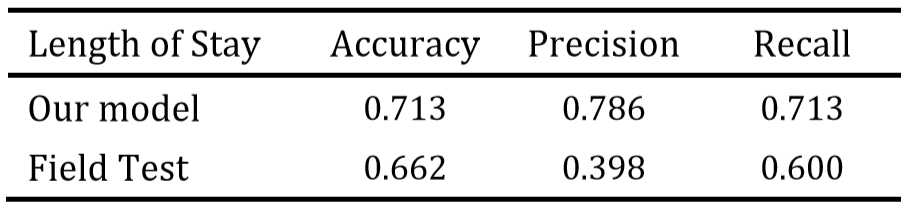

Supplement: Multimedia Appendix 15 [file medinform_v12i1e48862_app15.png]

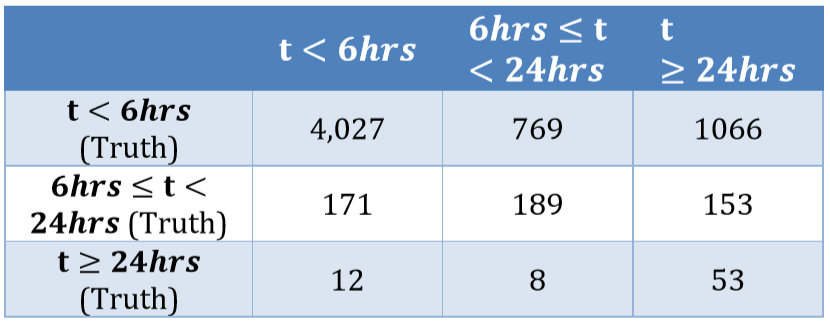

Supplement: Multimedia Appendix 16 [file medinform_v12i1e48862_app16.png]

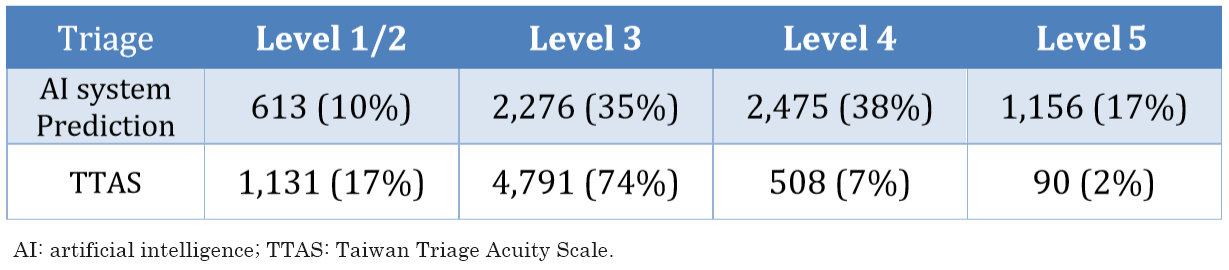

Supplement: Multimedia Appendix 17 [file medinform_v12i1e48862_app17.png]

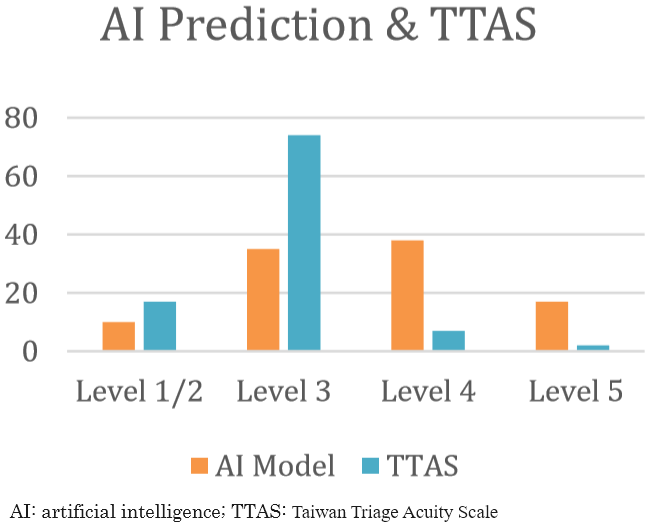

Supplement: Multimedia Appendix 18 [file medinform_v12i1e48862_app18.png]
